# Scale‐dependent effects of marine subsidies on the island biogeographic patterns of plants

**DOI:** 10.1002/ece3.9270

**Published:** 2022-09-09

**Authors:** Debora S. Obrist, Owen T. Fitzpatrick, Norah E. M. Brown, Patrick J. Hanly, Wiebe Nijland, Luba Y. Reshitnyk, Sara B. Wickham, Chris T. Darimont, John D. Reynolds, Brian M. Starzomski

**Affiliations:** ^1^ Earth to Ocean Research Group, Department of Biological Sciences Simon Fraser University Burnaby British Columbia Canada; ^2^ Hakai Institute Heriot Bay British Columbia Canada; ^3^ School of Environmental Studies University of Victoria Victoria British Columbia Canada; ^4^ Department of Fisheries and Wildlife Michigan State University East Lansing Michigan USA; ^5^ Department of Physical Geography Utrecht University Utrecht The Netherlands; ^6^ Department of Geography University of Victoria Victoria British Columbia Canada; ^7^ Raincoast Conservation Foundation Sidney British Columbia Canada

**Keywords:** biodiversity, island biogeography, marine‐derived nutrients, plant ecology, spatial scale, spatial subsidies

## Abstract

Although species richness can be determined by different mechanisms at different spatial scales, the role of scale in the effects of marine inputs on island biogeography has not been studied explicitly. Here, we evaluated the potential influence of island characteristics and marine inputs (seaweed wrack biomass and marine‐derived nitrogen in the soil) on plant species richness at both a local (plot) and regional (island) scale on 92 islands in British Columbia, Canada. We found that the effects of subsidies on species richness depend strongly on spatial scale. Despite detecting no effects of marine subsidies at the island scale, we found that as plot level subsidies increased, species richness decreased; plots with more marine‐derived nitrogen in the soil hosted fewer plant species. We found no effect of seaweed wrack at either scale. To identify potential mechanisms underlying the decrease in diversity, we fit a spatially explicit joint species distribution model to evaluate species level responses to marine subsidies and effects of biotic interactions among species. We found mixed evidence for competition for both light and nutrients, and cannot rule out an alternative mechanism; the observed decrease in species richness may be due to disturbances associated with animal‐mediated nutrient deposits, particularly those from North American river otters (*Lontra canadensis*). By evaluating the scale‐dependent effects of marine subsidies on island biogeographic patterns of plants and revealing likely mechanisms that act on community composition, we provide novel insights on the scale dependence of a fundamental ecological theory, and on the rarely examined links between marine and terrestrial ecosystems often bridged by animal vectors.

## Graphical abstract

Nutrients from the ocean affect plant diversity on land, but whether this effect can be detected depends on how closely you look. When we looked at plant diversity on entire islands, we found no measurable effect of marine nutrients; however, when we took a closer look at 1 m^2^ plots, we found that plots with more marine nutrients hosted fewer plant species. It is possible that river otter activity, one of the main sources of nutrients on these islands, causes disturbances that not all plant species can tolerate.

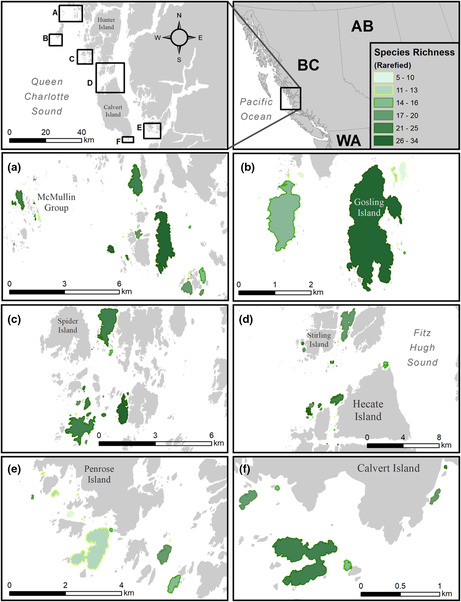

## 1 INTRODUCTION

The classical *theory of island biogeography* (TIB) proposed by MacArthur and Wilson (1967) predicts that the dynamic equilibrium of species richness on an island is a balance of immigration and extinction rates driven by isolation and island size. Due to its simplicity, this theory is widely applicable and thus has been highly influential (Whittaker et al., 2017). Since its inception, TIB has been modified and expanded to consider the additional roles of climate (Kalmar & Currie, 2006), habitat diversity (Ricklefs & Lovette, 1999), and invasive species (Blackburn et al., 2016), among others. Further modifications of TIB have led to the development of several related theories arising from more specific contexts. For instance, since in situ speciation is known to affect species diversity, particularly on oceanic islands, the *general dynamic theory* was developed to incorporate the additional influence of island age (Whittaker et al., 2008).

Through a phenomenon known as the *small island effect*, the species–area relationship often breaks down on small islands (Gao & Wang, 2022; Gentile & Argano, 2005; Heatwole & Levins, 1973; Morrison, 2014; Niering, 1963; Schrader et al., 2019). As such, breakpoint species–area models were established to allow species richness to vary independently from area on small islands (Lomolino & Weiser, 2001). The *subsidized island biogeography hypothesis* (SIB) is yet another modification of TIB, which was proposed to evaluate a potential mechanism behind the *small island effect* (Anderson & Wait, 2001). SIB considers the effects of nutrients, detritus, and organisms, which cross the boundary between marine and terrestrial ecosystems and have the potential to affect the densities of island species (Polis & Hurd, 1996). Due to our limited understanding of processes shaping ecosystems at the land–sea interface (Álvarez‐Romero et al., 2011), SIB is a particularly important addition to TIB.

Rather than considering islands as isolated entities, SIB builds on the classical TIB framework by proposing that inputs from the marine matrix surrounding small islands affect their terrestrial productivity (Anderson & Wait, 2001). Such inputs can be passive (i.e., associated with abiotic forces, including wind and wave action; Polis & Hurd, 1996) or active (i.e., animal‐mediated; McInturf et al., 2019). SIB assumes a unimodal relationship between productivity and diversity. Although this hump‐shaped relationship has been the subject of debate in the plant literature (Adler et al., 2011; Grace et al., 2016; Waide et al., 1999), on scales smaller than entire continents, this pattern is common in many vascular plant communities (Mittelbach et al., 2001). As marine inputs may facilitate higher productivity and therefore greater resource availability on nutrient‐poor islands, SIB posits that more species can co‐occur, resulting in an increase in both species densities and species diversity on subsidized islands. However, at higher rates of productivity derived from subsidies, some species may become competitively dominant, leading to a decrease in species diversity. According to SIB, small islands are expected to experience higher per‐unit area effects of marine inputs due to their higher perimeter–area ratios (i.e., more of the island is close to shore), providing a potential mechanism for the *small island effect* (Anderson & Wait, 2001).

Empirical tests of SIB have been few and have yielded mixed results. In the Bahamas, seabird presence had no effect on the lizard species richness–area curve (Barrett et al., 2003). Likewise, marine productivity had no observed effect on angiosperm diversity at the global level (Menegotto et al., 2019). In contrast, on temperate islands in coastal Canada, terrestrial birds were found in higher densities but with lower species richness on islands with higher levels of animal‐mediated subsidies (Obrist et al., 2020). These variable results are not surprising, given the context‐dependent nature of spatial subsidies (Subalusky & Post, 2019). However, determining the drivers of these variable effects is an important next step to improve our understanding of the meta‐ecosystem that encompasses the land–sea interface (Loreau et al., 2003). Indeed, despite coastal regions (including islands) hosting both disproportionately high degrees of human impacts (Williams et al., 2021) and contributions to biodiversity (Ray, 1991), our understanding of cross‐boundary processes at various scales at the land–sea interface remains limited (Fang et al., 2018).

Species richness on islands is determined by different mechanisms at different spatial scales (Rosenzweig & Ziv, 1999; Whittaker et al., 2001), yet the influence of marine subsidies on island biogeography has not been elaborated beyond the scale of the entire island (Anderson & Wait, 2001). At smaller spatial scales, such as sampling plots or transects on islands, species richness is typically determined by local environmental factors, stochastic events, biotic interactions, and regional species richness (Ibanez et al., 2018; Karger et al., 2014; MacArthur & Wilson, 1967; Schrader et al., 2019; Weigand et al., 2020). At larger spatial scales, such as at the level of entire islands, species richness is influenced by island area, isolation, habitat diversity, island age (Ibanez et al., 2018; Schrader et al., 2019), and even climate at the global scale (Menegotto et al., 2019; Weigelt & Kreft, 2013). Indeed, species richness is known to be dependent on spatial scale (Whittaker et al., 2001); as such, shedding light on the role of scale in determining the effects of marine inputs on island biogeography is important for understanding how island communities are assembled.

In this study, we investigate the role of spatial scale in subsidized island biogeography by evaluating the effects of marine subsidies on plant island biogeography at both a local (sampling plot) and regional (entire island) level. We conducted plant surveys on 92 islands in Haíɫzaqv and Wuikinuxv First Nation territories on the central coast of British Columbia, Canada. We use a series of hierarchical models to test the effects of classical TIB predictors (island area and isolation) and marine inputs at both spatial scales. We consider two metrics of marine inputs: shore‐cast macroalgal (wrack) biomass and marine‐derived nitrogen (δ^15^N) in the soil. By depositing materials containing the heavy isotope of nitrogen, ^15^N, marine subsidies often elevate soil δ^15^N in coastal terrestrial ecosystems (Ben‐David, Bowyer, et al., 1998; Ben‐David, Hanley, & Schell, 1998; Feddern et al., 2019). On the studied islands, marine subsidies likely come from many different sources (Obrist et al., 2022), including deposits of feces, urine, and discarded prey items of North American river otters (*Lontra canadensis*; Ben‐David, Bowyer, et al., 1998, C. Ernst, *unpublished data*), wind and wave‐deposited seaweed wrack (Wickham et al., 2020), sea spray (Weathers & Likens, 1997), and marine fog (Art et al., 1974). In our island level analysis, we also consider the potential effects of island slope, while in the plot level analysis, we consider soil moisture, plot slope, forest openness, and distance to shore. We further investigate which species might be driving patterns in plot level species composition by fitting a spatially explicit joint species distribution model (JSDM), which allows us to examine the underlying mechanisms on a species‐by‐species basis. Our comprehensive approach yields novel insights, both on the scale dependence of a fundamental ecological theory and on the understudied connections between marine and terrestrial ecosystems.

## 2 METHODS

### 2.1 Site description

**FIGURE 1:**
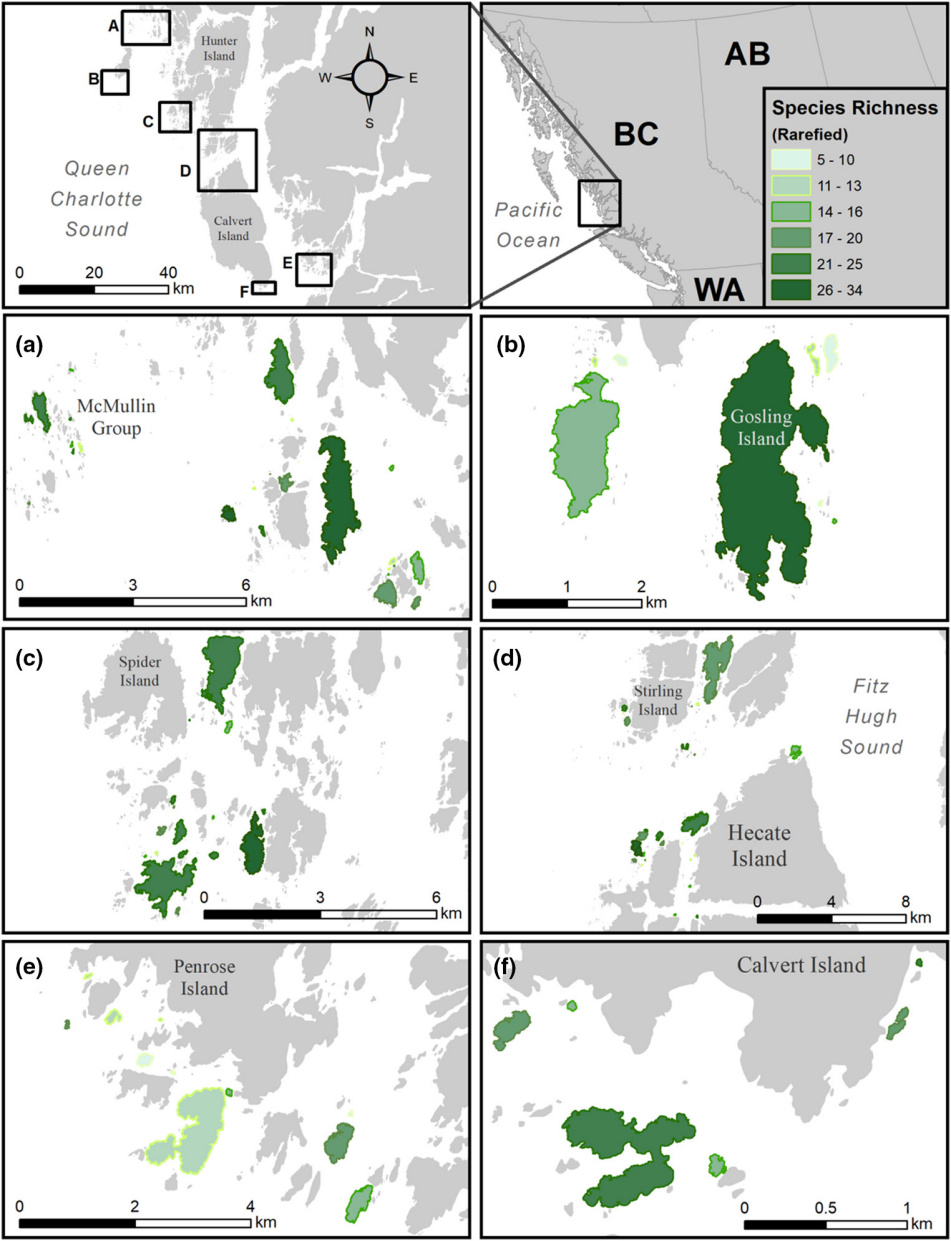
Study region on the central coast of British Columbia, Canada. Insets show the nine island nodes: (a) McMullin, Tribal, and Admiral, (b) Goose, (c) Triquet, (d) Stirling and Calvert, (e) Penrose, and (f) South Calvert. Sampled islands are highlighted in green, with deeper shades corresponding to higher species richness (*n* = 92).

We sampled plant communities on 92 islands ranging from 124 m^2^ to 3 km^2^ on the central coast of British Columbia, Canada, in the summers of 2015, 2016, and 2017 (Figure 1). This region is located within the hypermaritime subzone of the Coastal Western Hemlock biogeoclimatic zone (Banner et al., 1993). The climate is moderated by the influence of the Pacific Ocean, with mild winters, cool summers, abundant rainfall (>3 m per year; Pojar et al., 1987), and low evapotranspiration potential. Although nutrient‐limited (Miller, 2019), these islands are much more productive than the desert islands on which foundational work on SIB was conducted.

Study islands were selected from 1470 candidates using two‐step cluster analysis in SPSS (Corp, 2015). We generated a set of five descriptors: distance from mainland, area, normalized perimeter‐to‐area ratio, wave exposure, and the proportion of area occupied by land within a 500 m radius. We identified five clusters of divergent island types based on the set of descriptors. To facilitate sampling logistics, islands were then grouped by geographic proximity, where each geographic group contained islands from multiple cluster groups (Figure 1).

### 2.2 Field Sampling

**FIGURE A1:**
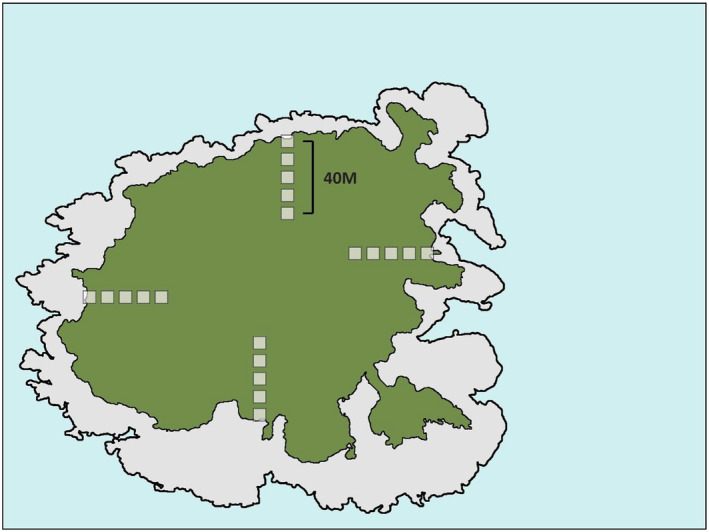
Layout for the field‐based observational sampling. Transects were established at each of the four cardinal directions. 1 m^2^ plots were spaced 10 m apart along transects that extended 40 m, from the shoreline towards the interior of islands. Soil samples were collected at the shoreline and interior plots on each transect.

On every island, we established a transect at each of four waypoints intersecting with shoreline at the four cardinal directions (Figure A1). Transects were perpendicular to the shoreline and extended 40 m into the interior of the island, although this distance decreased on islands that were <80 m wide (see details in Appendix A). We established five 1 m^2^ quadrats at 10 m intervals along each transect, starting at the shoreline. The shoreline plot was placed as close to the upper edge of the intertidal zone as possible, with the criterion that most of the plot's substrate was soil, and not solid rock, water, or other substrates unsuitable for plant growth.

#### 2.2.1 Plant surveys

**FIGURE A2:**
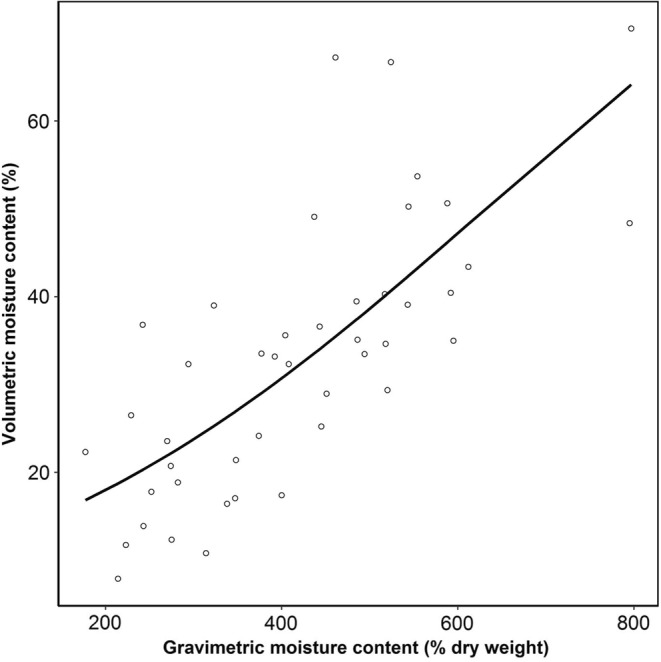
Volumetric moisture content, measured by the TDR 300 Soil Moisture Meter, increases with gravimetric moisture content expressed as % of dry soil weight (*n* = 44). Predicted line is derived from a beta regression model (*β* = 0.0035, *p* < .001, pseudo‐*R* ^2^ = 0.54, *n* = 44). The model was used to predict volumetric moisture content for plots that were not measured with the soil moisture meter.

In each quadrat, we measured percent cover of plant species (Table A1). We identified vascular plants to species, if possible, while both bryophyte (moss) and lichen cover were recorded as single estimates. We measured percent slope of each quadrat using a clinometer and took three volumetric soil moisture subsamples within each quadrat using a Field Scout TDR 300 Soil Moisture Meter. The soil moisture probe was not functioning for six islands (*n* = 97 quadrats); for these quadrats, we collected soil samples and imputed the missing volumetric values using a regression equation derived from plots with both volumetric and gravimetric soil moisture (Figure A2).

#### 2.2.2 Island characteristics

We derived estimates for island area and distance to the nearest vegetated landmass (our metric for isolation) using WorldView‐2 satellite imagery with 2 m resolution aquired from DigitalGlobe. Tidal and unvegetated areas were not included in the area calculation. The nearest vegetated landmass could be mainland or an island of any size. We also considered using the areas of surrounding landmasses within 250 m as a metric for isolation, as recommended by Weigelt and Kreft (2013). However, upon evaluation using Akaike's information criterion corrected for small sample sizes (AICc), we determined that there was no difference in explanatory power between these two models (i.e., ΔAICc <2, Burnham et al., 2010). As such, we continued to use distance to nearest vegetated landmass as an isolation metric. Terrain models (0.5 m resolution) were created for each island from lidar data and surveys using unmanned aerial vehicles (UAV, Nijland et al., 2017). We used these models to derive mean slope as the slope of the entire island, including the shore zone.

#### 2.2.3 Forest structure

We used the terrain models to derive estimates of forest structure variables. We created plot level forest structure variables in 10 m^2^ grid cells centered on each 1 m^2^ quadrat. The forest structure variables included estimates of tree height (mean height, max height, and volume) and canopy complexity (surface area ratio and surface volume ratio). These variables were reduced using principal components analysis (PCA), and scores from the first principal axis (PC1) were used as a single forest structure variable. PC1 explained 69% of the variation in the individual forest structure variables. Low forest structure PC1 scores were associated with taller, more structurally complex forests with higher basal area and canopy cover. This variable is henceforth called “forest openness”. See Appendix A for further detail on the ordination of forest structure variables.

#### 2.2.4 Marine inputs

We quantified marine inputs in two ways: (1) by weighing shore‐cast macroalgal biomass and (2) by measuring marine‐derived nutrients (specifically nitrogen) in the soil. We measured wrack biomass at shoreline sites centered on the cardinal direction waypoints (i.e., the transect start‐points) on each island. Two 20 m transects were established at each waypoint: one at the most recent high tide line, and one at the highest wrack line visible. We randomly selected three quadrats along each transect, where we measured the wet weight of each species, and converted to dry weights using Wickham et al. (2019)’s calibrations. In our plot level and island level analyses, we calculated wrack biomass as the mean amount of wrack (g) per site (two transects) and island, respectively. To measure inputs of marine‐derived nutrients to the terrestrial ecosystem, including those from river otter activity, sea spray, marine fog, and decomposing wrack biomass, we sampled soils at shoreline (0 m) and interior (40 m) quadrats of each transect (Appendix A). Soil δ^15^N is affected by both denitrification rates and marine subsidy inputs. In the denitrification process, soil microbes transform nitrate into gaseous N, a process which discriminates against ^15^N, resulting in enriched N pools in the soil (Pinay et al., 2003). Denitrification potential increases with nitrogen addition and soil moisture, which are affected by drainage and slope position (Bilby et al., 2003; Davidson & Swank, 1986). We sampled 250–500 g of soil from the first 10 cm of soil, with the litter layer removed. Percent soil nitrogen (%N) was measured using combustion elemental analysis and was expressed as a percentage of total soil mass (g/100 g). Soil δ^15^N was expressed in units of parts per mil (‰). Percent nitrogen and nitrogen stable isotope analyses were conducted at the Government of British Columbia's Analytical Chemistry Laboratory, and the Pacific Forestry Center, respectively.

### 2.3 Species richness

We identified 100 species of vascular plants in the 1 m^2^ quadrats on the 92 islands we sampled (Appendix A). Island scale rarefied species richness (sample‐based) ranged from approximately 5 to 34 species (Figure 1), whereas raw species richness (i.e., the number of species in all plots on a given island) ranged from 5 to 54 species. To compare plant species richness among islands, we performed sample‐based rarefaction and extrapolation with the *iNEXT* package in R version 3.6.3 (Hsieh, Ma and Chao, 2016; R Core Team, 2018), while our plot level species richness response is simply a count of the number of species observed in each 1 × 1 m plot. More details about the rarefaction methods are found in Appendix A.

### 2.4 Statistical analyses

#### 2.4.1 Island level species richness

To investigate drivers of island level rarefied species richness, we fit a global linear mixed effects model (LMM) with a Gaussian probability distribution to data from 92 islands using the *glmmTMB* package in R version 4.1.1 (Brooks et al., 2017; R Core Team, 2021). This global model included island area (m^2^), wrack biomass (kg/m^2^), forest‐edge soil δ^15^N (‰), the mean slope of the island (°), distance to the nearest vegetated landmass (m), and interactions between island area and both metrics of marine subsidies—forest‐edge soil δ^15^N and wrack biomass. To account for potential variation that could arise from sampling islands over different sampling periods and across different geographic groups of islands, we included “node” as a random effect. We model‐averaged across all possible subsets of predictors and the two interaction terms to obtain average coefficient estimates using the *MuMIn* package (Barton, 2020). We log_10_‐transformed island area and square root‐transformed wrack biomass to linearize their relationships with species richness, and we scaled and centered all independent variables. We used the *DHARMa* and *performance* packages to check model diagnostics (Hartig, 2020; Lüdecke et al., 2021). We checked variance inflation factors (VIFs) to assess multicollinearity between predictors (Zuur et al., 2009). The highest VIF was for soil δ^15^N (VIF = 2.2). We present a table of correlations between covariates in Table A2. We displayed model‐averaged coefficients in the figures but based our predictions on the global model coefficients (Burnham & Anderson, 2016; Cade, 2015).

#### 2.4.2 Plot level species richness

To assess plot level species richness, we followed a similar process, but because our plot level richness response was simply a count of the number of species in a plot, we fit a global generalized linear mixed effects model (GLMM) with a Poisson probability distribution. This global model contained island level parameters for island area (m^2^) and distance to the nearest vegetated landmass (m). It also included some transect level data: the wrack biomass (kg/m^2^) on shore at the start of the transect, and an average of the soil %N and δ^15^N (‰) between 0 m and 40 m. We computed these averages so that we would not lose the data from the 10 m, 20 m, and 30 m plots, which did not have corresponding plot level nutrient data. Plot level variables included in this model were the plot's slope (%), soil moisture (%), distance to shore (m), and forest openness (PC1). We also included an interaction term between island area and distance to shore, given that the effect of island area could depend on a site's distance to shore. For instance, a hypothetical plot that is 5 m from shore on a circular island that is 10 m in diameter would likely experience more marine influence (including but not limited to fog, sea spray, wind, exposure, and nutrients) than one that is 100 m in diameter, since it is 5 m from shore in all directions. Finally, we also included a nested random effect to account for the hierarchical nature of our sampling methods. As such, our random effect for the plot level analysis consisted of transect, nested within island, nested within island group (i.e., node). In this case, the highest VIF was for island area (VIF = 1.5). We present a table of correlations between covariates at the plot level in Table A3. Plots, transects, and islands with missing data were excluded from the plot level models; this resulted in a final sample of 1381 plots on 347 transects across 90 islands.

#### 2.4.3 Community composition

To analyze how island characteristics and marine subsidies might affect plant community composition and to evaluate the mechanism behind any patterns in species richness, we fit a spatially explicit joint species distribution model using the *Hmsc* package (Ovaskainen et al., 2017; Tikhonov et al., 2019, 2022; Tikhonov, Duan, et al., 2020; Tikhonov, Opedal, et al., 2020). We ran two Markov Chain Monte Carlo chains of 25,000 iterations, thinned to retain every 5th sample, and set to remove (burn‐in) the first 1000 iterations. We checked mixing by evaluating estimated sample size and potential scale reduction factors, and report root mean square errors (RMSE) and proportion of variance explained (*R* ^2^) and evaluated model fit through four‐fold cross validation. Specific results and diagnostics are in Appendix A (Table A4).

## 3 RESULTS

### 3.1 Island scale plant diversity

**FIGURE 2:**
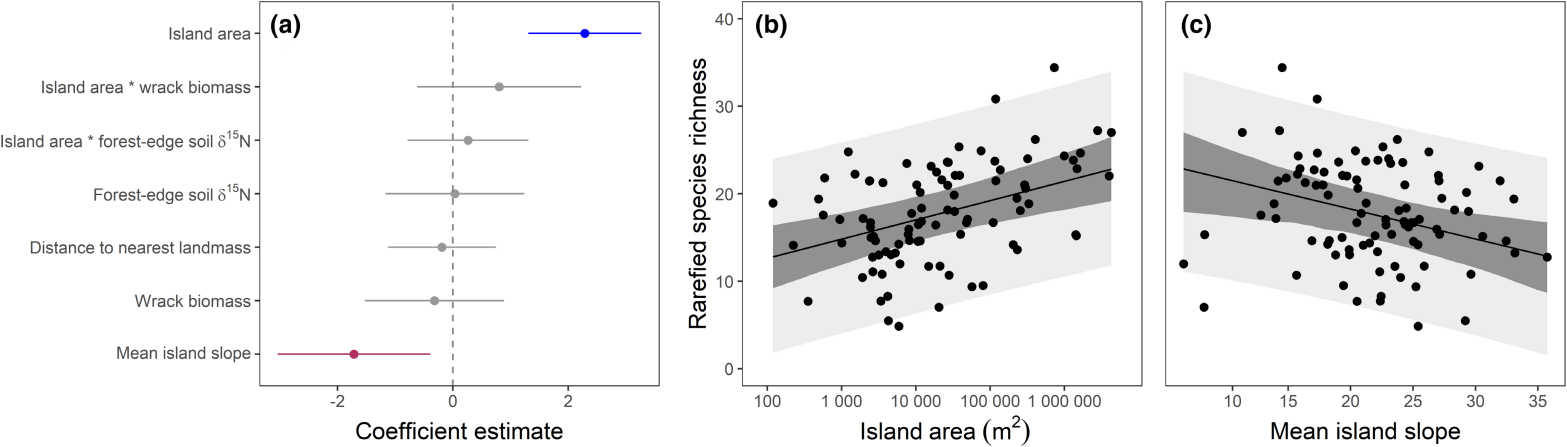
Model‐averaged coefficient estimates of island level rarefied plant species richness (a), and the modeled relationship with island area (b) and mean island slope (c) plotted over raw data. Lines in a and dark gray shading in b represent 95% confidence intervals. Light gray shading represents 95% prediction intervals.

We found that both area and mean slope of islands affect plant species richness on the island level. As predicted, island area was positively associated with species richness (Figure 1a,b). We found islands of median size (~13,000 m^2^) to have an average of 17.4 ± 2.5 (global model estimate ±95% confidence interval) plant species, while islands one order of magnitude larger (~130,000 m^2^) and smaller (~1300 m^2^) have 13% more (19.7 ± 2.8) and 15% fewer (15.1 ± 2.8) species of plant, respectively. We also found that steeper islands had fewer species on them—a flatter island with a mean slope of 15 degrees had 36% more species (19.5 ± 3.0) than one with a mean slope of 30 degrees (14.3 ± 3.3 species) (Figure 2a,c). The strength of the effect of island area and mean island slope were approximately equal. We found no evidence that forest‐edge soil δ^15^N, wrack biomass, or distance to nearest vegetated landmass had any effect on species richness at the island scale (Figure 2a). We also found no evidence of an interaction between island area and wrack biomass or between island area and forest edge soil δ^15^N.

### 3.2 Plot scale plant diversity

**FIGURE 3:**
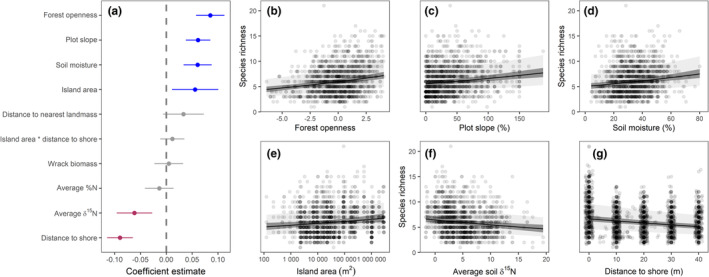
Model‐averaged coefficient estimates of plot level plant species richness (a), and the modeled relationship with forest openness (b), plot slope (c), soil moisture (d), island area (e), forest‐edge soil δ^15^N (f), and distance to shore (g), plotted over raw data. Lines in a and dark gray shading in b–g represent 95% confidence intervals. Light gray shading represents 95% prediction intervals.

At the 1 × 1 m plot level, factors affecting plant species richness are more complex (Figure 3). Plots in more open forests (Figure 3b) with steeper (plot) slopes (Figure 3c), those with higher soil moisture (Figure 3d), and those on larger islands had more species (Figure 3e). These four parameters were similar in strength, though island area carried more uncertainty. On larger islands (~1,300,000 m^2^), we estimated 13% more species, with an average of 6.3 ± 0.6 species per plot, while smaller ones would host 5.6 ± 0.5. In contrast, we found that plots with a higher average soil δ^15^N (Figure 3f), and plots further from shore (Figure 3g) had fewer species in them. Holding all else constant, we estimated shore‐side plots to have 43% more species than those 40 m inland (i.e., 6.7 ± 0.6 at shore vs 4.7 ± 0.5 40 m inland). In addition, a plot with 1 SD less than the median amount of marine‐derived nitrogen in the soil was estimated to have roughly 12% more species than one with 1 SD more than the median amount (i.e., 6.3 ± 0.6 as opposed to 5.6 ± 0.5 species). As with the island scale, we found no effect of wrack biomass on plot scale plant species richness.

### 3.3 Community composition

**FIGURE 4:**
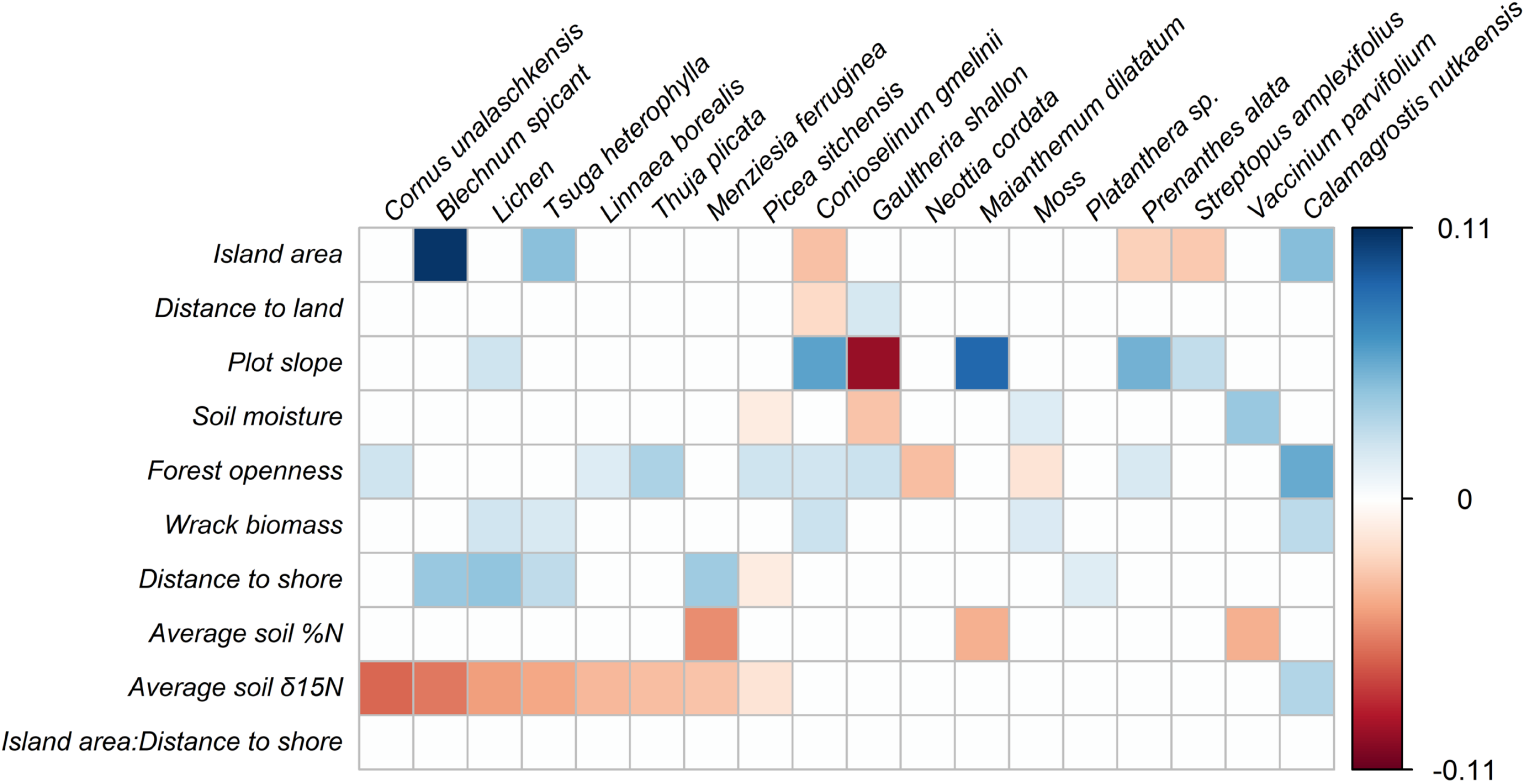
Plant species level responses to environmental parameters (posterior means) on 90 islands on the central coast of British Columbia, Canada. This plot shows estimates where the posterior probability of coefficients being negative or positive is greater than 95%. Positive responses (blue) indicate higher species abundances with higher values of the covariate on the y‐axis, while negative responses (red) indicate lower species abundances with higher values of the covariate.

As expected, plants had varied habitat preferences (Figure 4). Three species occurred more often (i.e., were positively associated) and three species occurred less often (i.e., were negatively associated) with larger islands. Eight of the 18 species were less abundant at sites with higher average soil δ^15^N, while one species, *Calamagrostis nutkaensis*, displayed a preference for these plots.

**FIGURE 5:**
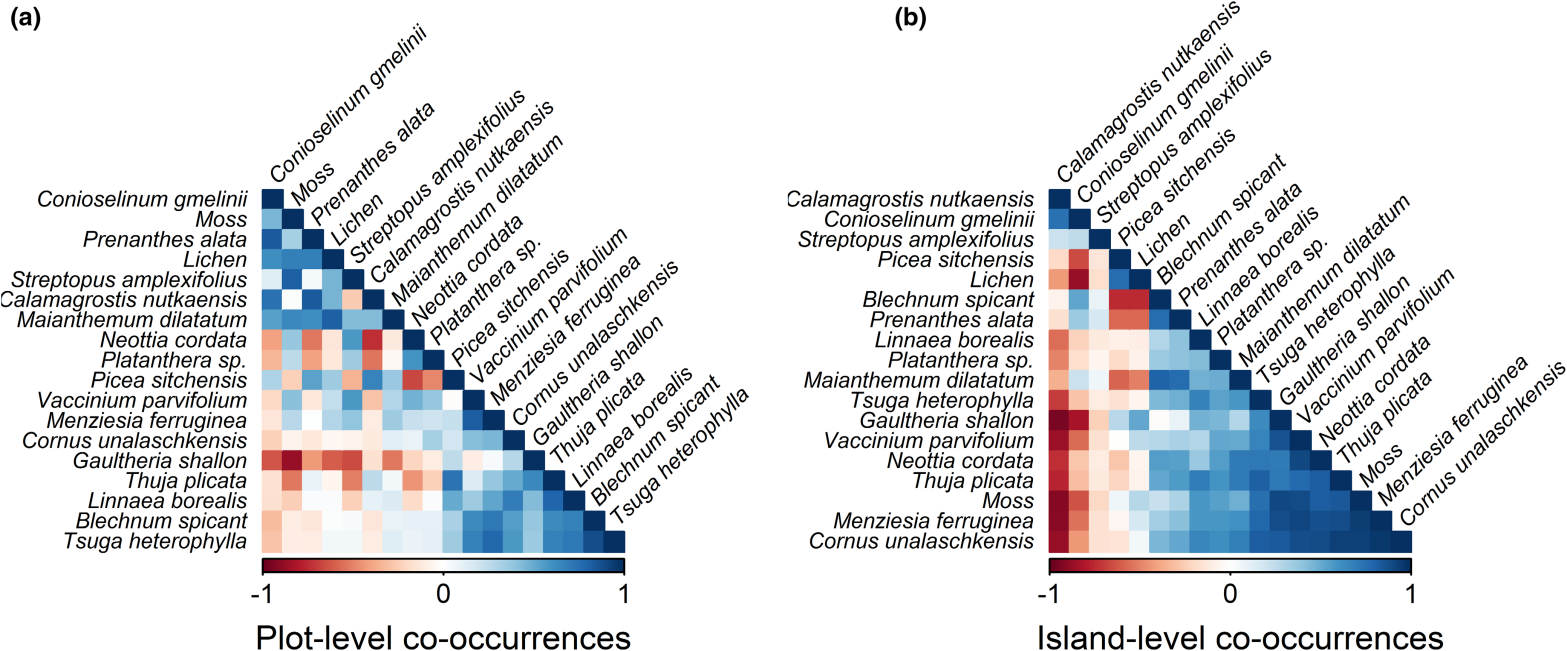
Plant species level co‐occurrences on 90 islands on the central coast of British Columbia, Canada at (a) the 1 × 1 m sampling plot level, and (b) co‐occurrences at the level of the entire island. Analyzes were run with the 18 plant species present in more than 5% of plots.

Finally, we found differences in patterns of species co‐occurrences at the plot versus island levels (Figure 5a,b). For example, the most abundant plant, *Gaultheria shallon*, had many negative associations at the plot level, but showed mostly positive associations at the island level. The second most abundant plant, *Maianthemum dilatatum*, displayed a strong negative plot level association with *G. shallon*. However, on the island level, these two species were positively associated. *M. dilatatum* displayed negative associations with different species: *C. nutkaensis*, *Picea sitchensis*, and with lichens. *C. nutkaensis*, the only species to display a preference for sites with higher average soil δ^15^N, displayed several negative co‐occurrences with other plant species at both the plot level and the island level; however, these negative co‐occurrences are stronger at the island level.

## 4 DISCUSSION

In this novel test of the scale dependence of marine inputs on island biogeography, we found that the effects of marine inputs on plant species richness depend on the spatial scale of investigation. At regional (island) scales, the effects of marine inputs were undetectable, while on the scale of the sampling plot, we found a decrease in plant species richness with more marine input. Specifically, we found that 1 × 1 m plots on transects with higher levels of δ^15^N in the soil hosted fewer plant species. Furthermore, we found mixed support for competition as the underlying mechanism behind this decrease in species richness, suggesting that the variable abilities of plants to compete for light and to tolerate the disturbances caused by the most likely sources of these subsidies—sea spray and river otter activity—may also play a role. Despite documented effects of wrack biomass on dune plant communities (e.g., Del Vecchio et al., 2017), we found no effect of wrack biomass on coastal plant species richness at either scale.

Although island area affected species richness at both local (plot) and regional (island) scales, we were only able to detect an effect of subsidies at the local scale. Since marine subsidies have localized, heterogeneous effects on environments (Davis & Keppel, 2021), it is possible that effects of subsidies “average out” over the scale of entire islands (Stein et al., 2014). Similarly, a study on trees in the Raja Ampat archipelago, West Papua Province, Indonesia found that island area affected both plot and island species richness, but habitat quality was far more important at local scales (Schrader et al., 2019). Likewise, the strength of the effect of area on fern species richness on a different set of Southeast Asian islands increased with spatial scale, while environmental conditions (i.e., plot slope, soil fertility, and canopy cover) were most important at local scales (Karger et al., 2014). In the current study, such local environmental characteristics were also more important in shaping plant communities than island area; forest openness, plot slope, soil moisture, and distance to shore all had stronger standardized effect sizes than island area at the plot scale. As such, our finding that a spatially heterogeneous environmental parameter is more important at smaller spatial scales is not unexpected, but our finding that marine subsidies do influence plant species richness at local scales provides an initial step towards filling the gap in knowledge about the role of spatial subsidies at the land–sea interface.

There are several possible explanations for a negative relationship between marine subsidies and plant diversity such as the one we observed. First, if marine inputs increase productivity on the studied islands, plant communities may fall on the downward‐sloping side of the productivity‐diversity curve, where, according to the *subsidized island biogeography hypothesis* (SIB), species richness may decrease as a result of increased interspecific competition and subsequent increased extinction rates for species unable to compete (Anderson & Wait, 2001). However, this hypothesis is unlikely because although our studied islands are relatively nutrient‐rich compared with the desert islands where SIB was conceived, soils on islands in this study are still nitrogen‐limited (Miller, 2019). Accordingly, we expect that nutrient inputs should yield increases in plant diversity. A second hypothesis suggests that fertilization decreases the amount of limiting resources in an ecosystem, effectively minimizing trade‐off opportunities for plants allowing for coexistence (Harpole et al., 2016). Furthermore, Hautier et al. (2009) suggest that through increased productivity, fertilization increases competition for light. Given that forest openness was the strongest driver of plant species richness at the plot scale, it is likely that competition for light impacts plant communities in our study. Dickson and Foster (2011), however, found that competition for light and fertilization are independent, additive processes, which makes it difficult to disentangle their potential contributions. Finally, given the variable tolerance of plants to disturbance by river otter‐mediated fertilization on coastlines in Alaska, it is also possible that species richness of plants on our studied islands decreased with increased fertilization as a response to physical disturbance (Ben‐David, Bowyer, et al., 1998; Roe et al., 2010).

In evaluating each of the above hypotheses, we infer that both competition for light and plant species' responses to the nature of river otter‐mediated fertilization likely play a role in decreasing plot level species richness on the studied islands. Competition for light seems likely; eight species showed a preference for sites with higher forest openness, and several of them showed negative associations with one another at the plot level, implying local competition. For instance, *Gaultheria shallon*, a thick, perennial shrub that dominates nutrient‐poor sites (Pojar & MacKinnon, 2004), displays negative co‐occurrences with all but three other species at the plot level. Lack of tolerance of river‐otter mediated fertilization is also a possibility; we found that eight species display negative associations with soil δ^15^N, rather than positive associations we would expect to see if the primary mechanism was competition for nutrients. Indeed, only the grass *Calamagrostis nutkaensis* showed a preference for sites with higher levels of soil δ^15^N. The natural history of *C. nutkaensis* makes it difficult to discern whether its negative co‐occurrences with several species at the plot level exemplify competition for nutrients or tolerance for harsh conditions. This species is known to tolerate wind exposure and salt spray (Pojar & MacKinnon, 2004) and is able to resprout vigorously from underground rhizomes postdisturbance (Sawyer, 2009). However, it is also often dominant in coastal ecosystems and is a good competitor against invasive species (Thomsen & D'Antonio, 2007). As such, our evidence for competition is mixed. Although we find evidence of plants competing for light, competition for nutrients is less clear, and we cannot discern whether additional nutrients result in increased competition for light (as suggested by Hautier et al., 2009). Additionally, it remains unclear whether species richness decreases with increases in soil δ^15^N because plant species cannot tolerate disturbances caused by the subsidy source, or if *C. nutkaensis* is outcompeting other species on a local scale.

**FIGURE A4:**
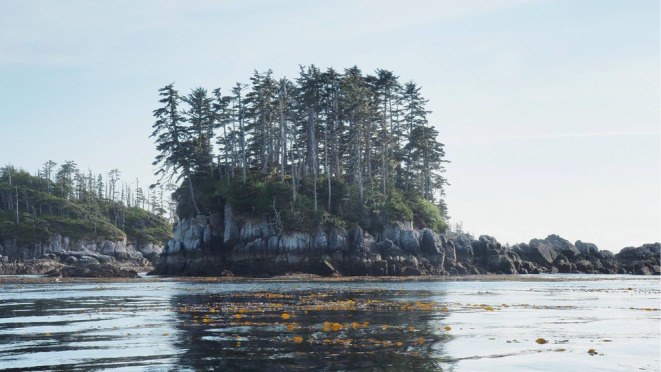
One of the studied islands in the Triquet node (Figure 1). Steep shoreline topography and surrounding kelp beds are characteristic of the islands in this study. Photo by Kate Prince.

Finally, despite previously finding higher wrack biomass corresponding to ^15^N enrichment in two plant species on the same set of islands (Obrist et al., 2022), we found no evidence of wrack affecting patterns in plant species diversity at the plot scale nor at the island scale. Given the high productivity of kelp forests in the study area (Steneck et al., 2002; Wilmers et al., 2012), we initially thought that wrack would be one of the main contributions of marine inputs to the islands in our study. However, we also found no effects of wrack on terrestrial breeding bird diversity or density here (Obrist et al., 2020). Wrack has been shown to be an important marine subsidy in several systems, including coastal dune vegetation in Sardinia (Del Vecchio et al., 2017), macrofauna and shorebirds on Californian beaches (Dugan et al., 2003), shorebirds on Australian beaches (Davis & Keppel, 2021) and plants, arthropods, and lizards in the Bahamas (Spiller et al., 2010). A commonality between these studies is one which our study system lacks: sandy beaches (see Figure A4 for a geographically representative island from the Triquet node). About 75% of wrack measurement sites in our study consisted of rock substrate (Wickham et al., 2020), and islands tended to be steep, with a mean overall slope (including the shore zone) of 21°. Substrate type and shoreline slope are important determinants of wrack retention, with steep shorelines and rocky substrates retaining significantly less wrack than sand, cobble, or boulder beaches (Orr et al., 2005; Wickham et al., 2020). As such, the potential signal of wrack effects on terrestrial plant communities may be overshadowed by subsidy sources not impeded by rocky shorelines, such as those contributed by animal vectors.

## 5 CONCLUSION

We found evidence for scale‐dependent effects of marine inputs. Marine subsidies affected plant species richness on local but not regional scales on the 92 islands that we studied. This finding demonstrates the importance of understanding the scale at which cross‐boundary transfers subsidize ecosystems. Furthermore, our finding that the source of subsidy may interact with or even counteract nutrient benefits demonstrates that many facets can contribute to the community assembly of plant species on islands. This is particularly relevant in the context of animal‐mediated transfers that often bridge the land–sea interface between marine and terrestrial ecosystems, a system that has been considerably understudied.

## AUTHOR CONTRIBUTIONS

**Debora S. Obrist:** Conceptualization (equal); data curation (supporting); formal analysis (equal); writing – review and editing (lead). **Owen T. Fitzpatrick:** Conceptualization (equal); data curation (lead); formal analysis (equal); writing – original draft (lead). **Norah Brown:** Formal analysis (supporting); writing – review and editing (supporting). **Patrick J. Hanly:** Formal analysis (supporting); writing – review and editing (supporting). **Wiebe Nijland:** Data curation (supporting); formal analysis (supporting); writing – review and editing (supporting). **Luba Y. Reshitnyk:** Data curation (supporting); writing – review and editing (supporting). **Sara B. Wickham:** Data curation (supporting); writing – review and editing (supporting). **Chris T. Darimont:** Conceptualization (equal); funding acquisition (equal); writing – review and editing (supporting). **John D. Reynolds:** Conceptualization (equal); funding acquisition (equal); writing – review and editing (supporting). **Brian M. Starzomski:** Conceptualization (equal); funding acquisition (equal); writing – review and editing (supporting).

## CONFLICT OF INTEREST

Authors declare no conflict of interest.

## Figures and Tables

**FIGURE A3:**
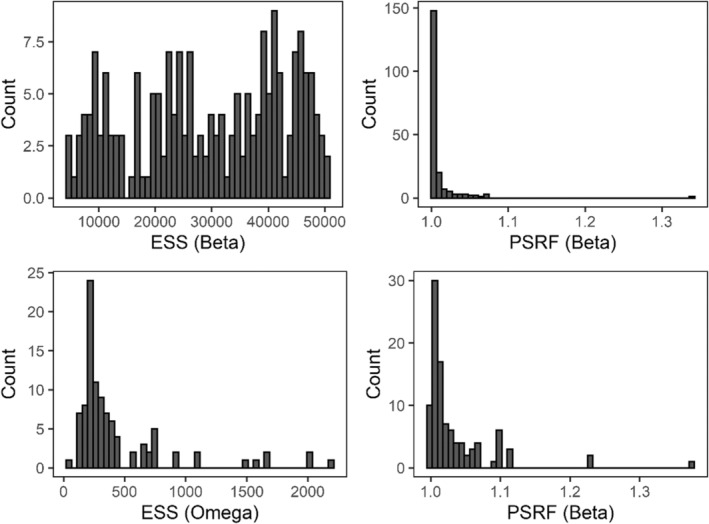
Effective sample size (ESS) and potential scale reduction factors (PSRF) can be used to quantitatively evaluate chain convergence in Markov Chain Monte Carlo sampling (Tikhonov et al., 2019, 2022). If ESS is similar to the theoretical number of samples, autocorrelation among consecutive samples is low. In this case, we evaluated 25000 samples in 2 chains (theoretical sample size = 50000). PSRF values close to 1 indicate that the two chains give similar results (i.e., have mixed well).

## Data Availability

Data for this work are archived in the Hakai Institute’s Metadata Catalogue: https://doi.org/10.21966/CZ48‐D388. It is executable with code from https://github.com/debobrist/plant‐sib.

## APPENDIX A

### Site description

Although all sampled islands were forested, shoreline edge communities varied widely. Larger islands contained bog woodland and open bog in their interiors. Typical forests of the region are relatively open and are dominated by *Thuja plicata* (western red cedar), *Chamaecyparis nootkatensis* (yellow cedar), and *Tsuga heterophylla* (western hemlock). Common shrub‐layer species include *Gaultheria shallon* (salal), *Vaccinium* spp including *V. ovalifolium* (oval‐leaved blueberry) and *V. parvifolium* (red huckleberry), and *Menziesia ferruginea* (false azalea). Common herb‐layer species include *Cornus unalaschkensis* (bunchberry), *Blechnum spicant* (deer fern), and *Maianthemum dilatatum* (false lily of the valley). The bogs of the interiors of larger islands include ericaceous shrubs such as *Kalmia microphylla* (bog‐laurel), sedges such as *Trichopohorum cespitosum* (tufted clubrush) and *Eriophorum angustifolium* (cotton‐grass), and other typical bog species (e.g., *Drosera rotundifolia*, round‐leaved sundew).

### Data collection

On 41 of 92 islands, we sampled exactly four transects, with five plots on each transect. However, on islands larger than 0.5 km^2^, we added transects (the number scaled with size), up to a maximum of four additional transects. In addition, on smaller islands (when the distance from a shoreline to an opposite shoreline was estimated to be 60 m or less) we established a single transect to span the island along that axis. For the smallest islands, we established four shoreline (0 m) quadrats, and as many interior quadrats as possible while maintaining a 10 m spacing between quadrats (see Figure A1 below). The 0 m quadrat was established as close as possible to the shoreline, with the criterion that the majority of the substrate was soil.

Unmanned aerial vehicle (UAV) and lidar data were used to generate several remotely‐sensed forest structure variables in 10 m^2^ grid cells surrounding each 1 m^2^ quadrat. These variables were measures of canopy height (mean and max canopy height, volume) and canopy complexity (surface volume ratio, and surface area ratio). *Volume* is related to canopy height metrics and is a measure of the volume under the canopy surface. *Canopy complexity* is a measure of the regularity of the canopy surface: that is, whether the canopy surface is even, or whether it is characterized by unequal canopy heights, gaps, etc. Greater complexity is thought to be associated with older stands (Lefsky et al., 1999). *Surface volume ratio* is the ratio of the volume under the canopy to the volume under a box that is the same height as the top of the canopy surface. *Surface area ratio* is the ratio of the surface area of the canopy to the surface area of an orthogonal, flat surface. This has also been called “rumple” and has been shown to be correlated with increasing stand age (Kane et al., 2010).

We used a principal components analysis (PCA) to derive a single variable representing forest structure at the plot scale. The first principal axis explained 69% of the variation in the individual forest structure variables and was negatively correlated with all variables representing the height and structural complexity of the overstory. Plot PC1 scores were also negatively correlated with field‐based forest structure metrics, which were collected using the point‐centered quarter method (Mitchell, 2015) centered on each plot: basal area, *r*(1548) = −0.33, *p* < .001; stem density, *r*(1548) = −.03, *p* < .01; and canopy cover, *r*(1002) = −0.38, *p* < .001. In other words, plots with lower combined “forest structure” (PC1) values were surrounded by taller and more structurally complex forests, with higher stem density, basal area and canopy cover.

### Soil moisture imputation

The Field Scout TDR 300 soil moisture probe was not functioning for a subset of six islands (*n* = 97 quadrats). We collected a soil sample at each of these quadrats and calculated the gravimetric moisture content following a standard protocol. With a working Field Scout TDR 300, we then measured both gravimetric and volumetric soil moisture for a set of quadrats (*n* = 44) and used a beta regression model based on those quadrats to predict volumetric soil moisture for the missing volumetric soil moisture values (*β* = 0.0035, *p* < .001, pseudo‐*R* ^2^ = 0.54; see Figure A2 below).

### Species richness and percent cover calculations

#### Species richness

We used slightly different metrics for species richness at the plot level and at the island level. Our plot‐level species richness response is simply a count of the number of species found in each 1 × 1 m plot. However, to compare plant species richness among islands, we performed sample‐based rarefaction and extrapolation with the “iNEXT” package (Hsieh et al., 2016) in R (v.3.6.3). To provide a more complete measure of island species richness, we used additional percent cover data from a concurrent project on the same islands. These 1 m^2^ quadrats were placed at avian point count locations, which were spaced at 250 m intervals and stratified by habitat type (Obrist et al., 2020). We converted the percent cover data of each quadrat to incidence data (presence/absence) for the rarefaction process. We standardized to 14 quadrats per island, below the median number of 20 quadrats per island. We selected 14 because it was twice the reference sample size of the smallest four islands (*n* = 7 quadrats), which is the most recommended factor to support reliable extrapolation (Chao et al., 2014).

Our measure of island‐scale species richness is thus the cumulative species richness per island in a standardized 14 m^2^ area. Some island biogeography studies use exhaustive surveys (e.g., Cody, 2006; Morrison, 1997) or systematic belt transects (e.g., Kohn & Walsh, 1994) to survey for species, whereas our quadrat‐based sampling design was a trade‐off to allow sampling on 92 very remote islands over 3 years and a more intensive examination of the possible influence of marine subsidies on plant communities—particularly at the shoreline edge.

#### Plot‐scale plant percent cover

Given that our plant cover data were collected over 3 years by several people, we had to account for some variation in data collection techniques. For example, in 1 year, only the total percent cover of all species at all heights was recorded, while in other years, covers were recorded per layer (ground (<10 cm), field (10 cm–50 cm), and shrub (50 cm–2 m)). We accounted for this using Fischer's (2015) formula to convert the covers of species in separate layers into one value for total cover for each species. This formula assumes independent overlap of layers and is denoted as

$$1-\prod_{l=1}^{n}\left(1-p_{1}\right),$$

where *n* is the number of layers of vegetation cover for each species in each plot, and *p* is the percent cover of a given species in the given layer.

### Community composition: HMSC model

This hierarchical modeling of species communities approach uses Bayesian inference to disentangle the effects of environmental parameters and species interactions on species abundances. It simultaneously estimates species' responses to a matrix of environmental parameters, for which we provided the same parameters as in the plot‐level species richness GLMM, across all samples. At the same time, it estimates species co‐occurrences through correlations in residuals. Since our species data were in the form of percent cover, we used a normal distribution. We incorporated a detailed nested random effects structure, considering that plots are nested within transects, and transects are nested within islands. We also included a spatial random effect, estimated by the latitude and longitude of each plot; however, since this proved to be very computationally intensive, we used the nearest‐neighbor Gaussian process (NNGP, Datta et al., 2016) to condition the nonindependence of sites on the nearest 10 neighboring sites rather than all surveyed sites. This technique balances the trade‐offs between computational time and predictive performance of the model (Tikhonov, Duan, et al., 2020). With “Hmsc”, we ensured adequate mixing of Markov Chain Monte Carlo chains by evaluating the effective sample size and potential scale reduction factors (see Figure A3 below). We checked model fit (*R* ^2^ and RMSE) for both the explanatory power of the model, and for predictive power using 4‐fold cross‐validation (See Table A1 below).

**TABLE A1:** Vascular plant species encountered on 92 islands on the Central Coast of B.C, in (a) 1584 1 m^2^ quadrats along 40 m transects starting at shoreline, and b) in 296 randomly sampled 1 m^2^ quadrats (only species not also found along the transects are listed here)

Haíɫzaqvḷa (Haíɫzaqv)	’Uik̓ala (Wuikinuxv)	Latin name	Common name	Family	Plot % Frequency (*n*/1584)	Island % Frequency (*n*/92)
**(a) Quadrats along 40 m transects (*n* = 1584)**						
–	–	*Achillea millefolium*	Yarrow	Asteraceae	0.95 (15)	14 (13)
yát̓ás	–	*Alnus viridis* ssp. *sinuata*	Sitka alder	Betulaceae	0.06 (1)	1 (1)
–	tiɫàs	*Amalanchier alnifolia*	Saskatoon	Rosaceae	0.06 (1)	1 (1)
–	–	*Angelica lucida*	Sea–watch	Apiaceae	4.3 (68)	30 (28)
–	–	*Aquilegia formosa*	Red columbine	Ranunculaceae	0.57 (9)	8 (7)
–	–	*Arabis eschscholtziana*	Escholtz's hairy rockcress	Brassicaceae	0.19 (3)	3 (3)
ƛ̓álákn	–	*Aruncus dioicus* var. *acuminatus*	Goatsbeard	Rosaceae	0.06 (1)	1 (1)
–	–	*Asplenium viride*	Green spleenwort	Aspleniaceae	0.51 (8)	6 (6)
–	–	*Athyrium filix‐femina*	Lady fern	Dryopteridaceae	1.2 (19)	14 (13)
k̓áláx	–	*Blechnum spicant*	Deer fern	Blechnaceae	18 (291)	51 (47)
–	–	*Boschniakia hookeri*	Vancouver groundcone	Orobanchaceae	0.25 (4)	4 (4)
–	–	*Calamagrostis nutkaensis*	Pacific reedgrass	Poaceae	14 (214)	71 (65)
–	–	*Calypso bulbosa*	Fairy‐slipper	Orchidaceae	0.13 (2)	2 (2)
–	–	*Cardamine oligosperma*	Little western bitter‐cress	Brassicaceae	0.06 (1)	1 (1)
–	–	*Carex lyngbyei*	Lyngbye's sedge	Cyperaceae	0.06 (1)	1 (1)
–	–	*Carex obnupta*	Slough sedge	Cyperaceae	0.76 (12)	8 (7)
–	–	*Carex pluriflora*	Many‐flowered sedge	Cyperaceae	0.63 (10)	9 (8)
–	–	*Carex sitchensis*	Sitka sedge	Cyperaceae	0.13 (2)	2 (2)
–	–	*Castilleja miniata*	Common red paintbrush	Orobanchaceae	2.7 (43)	29 (27)
díw̓ás	diìwas/ diìw̓as	*Chamaecyparis nootkatensis*	Yellow‐cedar	Cupressaceae	0.32 (5)	4 (4)
–	–	*Cladothamnus pyroliflorus*	Copperbush	Ericaceae	0.13 (2)	1 (1)
–	–	*Claytonia sibirica*	Siberian miner's‐lettuce	Montiaceae	0.06 (1)	1 (1)
–	ha‐tùm	*Conioselinum gmelinii*	Pacific hemlock‐parsley	Apiaceae	6.2 (98)	47 (43)
–	–	*Coptis asplenifolia*	Three‐leaved goldthread	Ranunculaceae	0.06 (1)	1 (1)
ƛṃ́k̓vúlí	ƛàƛq̓^w^as	*Cornus unalaschkensis*	Alaskan bunchberry	Cornaceae	23 (372)	59 (54)
–	–	*Dodecatheon pulchellum*	Few‐flowered shootingstar	Primulacaea	0.13 (2)	1 (1)
–	t̓ìbàm (fern root)	*Dryopteris expansa*	Spiny wood fern	Dryopteridaceae	1.5 (23)	17 (16)
–	–	*Empetrum nigrum*	Crowberry	Ericaceae	4.2 (66)	47 (43)
c̓ax̌m̓	c̓àx̌m	*Epilobium angustifolium*	Fireweed	Onagraceae	0.13 (2)	2 (2)
–	–	*Equisetum arvense*	Common horsetail	Equisetaceae	0.06 (1)	1 (1)
–	–	*Equisetum* spp.	Various	Equisetaceae	0.13 (2)	1 (1)
ƛ̓xƛ̓k̓s	qlúlu/ql̓ulux̌^w^/t̓hxt̓hxk̓s	*Fragaria chiloensis*	Coastal strawberry	Rosaceae	3 (48)	37 (34)
xvúk̓vás	x^w^ùk̓^w^as	*Fritillaria camschatcensis*	Northern rice‐root	Liliaceae	3.2 (50)	33 (30)
–	–	*Galium aparine*	Cleavers	Rubiaceae	0.25 (4)	4 (4)
–	–	*Galium trifidum*	Small bedstraw	Rubiaceae	0.38 (6)	5 (5)
–	–	*Galium triflorum*	Sweet‐scented bedstraw	Rubiaceae	0.13 (2)	2 (2)
nk̓vɫ	nk̓vàs	*Gaultheria shallon*	Salal	Ericaceae	88 (1388)	98 (90)
–	–	*Goodyera oblongifolia*	Rattlesnake‐plantain	Orchidaceae	0.32 (5)	2 (2)
–	–	*Juncus arcticus*	Arctic rush	Juncaceae	0.19 (3)	3 (3)
–	–	*Leymus mollis*	Dune wildrye	Poaceae	0.88 (14)	9 (8)
–	–	*Linnaea borealis*	Twinflower	Caprifoliaceae	7.7 (122)	28 (26)
–	–	*Neottia caurina*	Northwestern twayblade	Orchidaceae	2.5 (39)	24 (22)
–	–	*Neottia cordata*	Heart‐leaved twayblade	Orchidaceae	9.5 (150)	50 (46)
–	–	*Loiseleuria procumbens*	Alpine‐azalea	Ericaceae	0.06 (1)	1 (1)
–	k̓^w^t̓x̌ùli	*Lonicera involucrata*	Black twinberry	Caprifoliaceae	3.5 (56)	27 (25)
–	–	*Luzula multiflora*	Many‐flowered wood‐rush	Juncaceae	0.19 (3)	3 (3)
–	w̓úsigṃ yis_◡_qam̓ila	*Lycopodium clavatum*	Running club‐moss	Lycopodiaceae	0.13 (2)	1 (1)
k̓vk̓vúk̓v	k̓^w^uuk̓^w^	*Lysichiton americanus*	Skunk cabbage	Araceae	0.82 (13)	11 (10)
–	t̀ṃc̓	*Maianthemum dilatatum*	False lily of the valley	Asparagaceae	55 (879)	98 (90)
k̓vṃ́tkv	ɫṇx̌. ḿas /lhə̀nx̌əmm̓as	*Malus fusca*	Pacific crab apple	Rosaceae	2.7 (42)	32 (29)
–	ƛṇq̓^w^as	*Menziesia ferruginea*	False azalea	Ericaceae	30 (479)	85 (78)
–	–	*Mimulus guttatus*	Yellow monkey‐flower	Phrymaceae	0.57 (9)	8 (7)
–	–	*Moneses uniflora*	Single delight	Ericaceae	0.51 (8)	8 (7)
wíq̓ás	wìq̓as	*Oploplanax horridus*	Devil's club	Araliaceae	0.95 (15)	6 (6)
h̓ṇíw̓as	h̓ṇiw̓as	*Picea sitchensis*	Sitka spruce	Pinaceae	5.4 (85)	53 (49)
–	Wàdayu	*Pinguicula vulgaris*	Common butterwort	Lentibulariaceae	0.13 (2)	2 (2)
ƛ̓ín̓a	ƛ̓ìq̓^w^as	*Pinus contorta*	Shore pine	Pinaceae	0.25 (4)	3 (3)
–	–	*Plantago macrocarpa*	Alaska plantain	Orchidaceae	0.13 (2)	2 (2)
–	–	*Plantago maritima*	Seaside plantain	Plantagineacea	0.57 (9)	9 (8)
–	–	*Platanthera* spp.	Various	Plantaginaceae	6.4 (101)	35 (32)
–	–	*Platanthera stricta*	Slender rein orchid	Orchidaceae	0.51 (8)	6 (6)
k̓áláx	ƛ̓ùḱ^w^ṃ	*Polypodium glycyrrhiza*	Licorice fern	Polypodiaceae	3.8 (60)	37 (34)
k̓áláx	k̓alax/sal̓idana	*Polystichum munitum*	Sword fern	Dryopteridaceae	1.6 (25)	14 (13)
–	–	*Potentilla villosa*	Villous cinqefoil	Rosaceae	0.32 (5)	5 (5)
–	–	*Prenanthes alata*	Western rattlesnake‐root	Asteraceae	14 (224)	77 (71)
sa̓’gŭm (Compton)	sa̓’gŭm/sag^w^ṃ	*Pteridium aquilinum*	Bracken fern	Denstaedtiaceae	4.4 (69)	27 (25)
–	–	*Pyrola picta*	White‐veined wintergreen	Ericaceae	0.06 (1)	1 (1)
–	–	*Ranunculus occidentalis*	Western buttercup	Ranunculaceae	0.06 (1)	1 (1)
–	–	*Rhamnus purshiana*	Cascara	Rhamnaceae	0.82 (13)	8 (7)
púy̓ás	púy̓as	*Rhododendron groenlandicum*	Labrador tea	Ericaceae	0.44 (7)	6 (6)
–	tə̀mxvim̓as/tṃx^w^.m̓as	*Ribes divarticum*	Coastal black gooseberry	Grossulariaceae	0.06 (1)	1 (1)
–	cṇ̀̀yas/cnyas	*Ribes lacustre*	Black gooseberry	Grossulariaceae	0.44 (7)	4 (4)
básbúlí?as	bàsbuliỷas	*Rosa nutkana*	Nootka rose	Rosaceae	0.19 (3)	2 (2)
l̓qáx̌ḷ̓ás	l̓qàx̌ṃ̓as/l̓qax̌ṃ̓azis/l̓qàx̌ṃas	*Rubus parviflorus*	Thimbleberry	Rosaceae	0.51 (8)	3 (3)
–	ǧùx̌^w^ǧ^w^ļs	*Rubus pedatus*	Five‐leaved bramble	Rosaceae	0.32 (5)	3 (3)
ǧúláli	ǧùl̓as/ǧúlalì	*Rubus spectabilis*	Salmonberry	Rosaceae	4.1 (65)	28 (26)
–	–	*Sagina maxima*	Coastal pearlwort	Caryophyllaceae	0.95 (15)	15 (14)
–	–	*Sisyrinchium littorale*	Shore blue‐eyed grass	Iridaceae	1 (16)	14 (13)
–	–	*Stellaria crispa*	Crisp starwort	Caryophyllaceae	0.19 (3)	3 (3)
–	–	*Streptopus amplexifolius*	Clasping twistedstalk	Liliaceae	6.2 (98)	43 (40)
ƛ̓ṃ́q̓	ƛ̓ṃ́q̓as	*Taxus brevifolia*	Western yew	Taxaceae	1.3 (20)	14 (13)
dṇ́y̓ás	də̀ny̓as/dṇ́y̓as/dnyas	*Thuja plicata*	Western redcedar	Cupressaceae	12 (187)	63 (58)
–	–	*Tiarella trifoliata (*var. *trifoliata &* var. *laciniata)*	Three‐leaved foamflower, cut‐leaved foamflower	Saxifragaceae	3.3 (53)	22 (20)
–	–	*Triantha glutinosa*	Sticky false asphodel	Tofieldiaceae	0.06 (1)	1 (1)
–	–	*Trientalis europaea* subsp. *arctica*	Northern starflower	Myrsinaceae	3.3 (53)	41 (38)
lúq̓vás	lùq̓^w^as	*Tsuga heterophylla*	Western hemlock	Pinaceae	14 (224)	62 (57)
–	–	*Vaccinium caespitosum*	Dwarf blueberry	Ericaceae	0.06 (1)	1 (1)
siák̓vṇat	q̓^w^ìq^w^x^w^sṃ/q̓^w^ìxsṃ/sḷsa	*Vaccinium ovalifolium*	Oval‐leaved blueberry	Ericaceae	1.6 (25)	20 (18)
ǧvádṃ	ǧ^w^àdṃ/ḳwa‐ṭŭm/ǧ^w^àt̓as	*Vaccinium parvifolium*	Red huckleberry	Ericaceae	29 (453)	85 (78)
h̓a̓úx̌vsúlí	h̓áùx̌^w^suli	*Veratrum viride*	Green false hellebore	Melanthiaceae	0.13 (2)	2 (2)
–	–	*Vicea nigricans* ssp. *gigantea*	Giant vetch	Fabaceae	1.5 (23)	18 (17)
**(b) randomly sampled quadrats (*n* = 296)**						
–	–	*Coptis trifolia*	Threeleaf goldthread	Ranunculaceae		
–	–	*Drosera rotundifolia*	Round‐leaved sundew	Droseraceae		
–	–	*Gentiana douglasiana*	Swamp gentian	Gentianaceae		
–	–	*Kalmia microphylla*	Western bog‐laurel	Ericaceae		
ƛ̓ín̓a	–	*Lycopodium dendroideum*	Ground‐pine	Lycopodiaceae		
–	–	*Myrica gale*	Sweet gale	Myricaceae		
–	–	*Piperia unalascensis*	Alaska rein orchid	Orchidaceae		
–	–	*Plantathera dilatata*	White bog orchid	Orchidaceae		
ƛxsa̓ṃ́	ƛxsáṃ̓/tlíṅ‐sŭm/ƛk	*Potentilla anserina*	Silverweed	Rosaceae		
–	–	*Sanguisorba officinalis*	Great burnet	Rosaceae		
ƛ̓ṃsdáic̓	t̓ə̀ll̓c	*Vaccinium oxycoccos*	Bog cranberry	Ericaceae		

*Note*: Haíɫzaqvḷa names provided by William Housty (Heiltsuk Integrated Research Management Department),’Uik̓ala names are from Compton (1993). Latin nomenclature follows the *Illustrated Flora of British Columbia* (Douglas et al., 1998–2002). For each species encountered in 1 m^2^ quadrats along transects (i.e. excluding any species encountered in randomly sampled quadrats), their percent frequency both in quadrats (*n* = 1584) and on islands (*n* = 92) is presented. The raw count of occupied plots and islands is in brackets. Due to some missing variables, not all plots were included in our plot‐level analyzes.

**TABLE A2:** Correlations between parameters at the island level

	Island area	Wrack biomass	Forest‐edge soil δ^15^N	Mean island slope	Distance to nearest landmass
Island area	1.00	0.05	−0.62	−0.23	−0.12
Wrack biomass	0.05	1.00	−0.11	−0.51	0.04
Forest‐edge soil δ^15^N	−0.62	−0.11	1.00	0.22	0.33
Mean island slope	−0.23	−0.51	0.22	1.00	−0.04
Distance to nearest landmass	−0.12	0.04	0.33	−0.04	1.00

*Note*: Wrack biomass and forest‐edge soil δ15N are island level averages. Mean island slope includes the shore zone. Distance to nearest landmass is the distance from the island centroid to the nearest vegetated landmass of any size.

**TABLE A3:** Correlations between parameters at the plot level

	Island area	Dist. to shore	Dist. to landmass	Wrack biomass	Forest openness	Soil moisture	Plot slope	Avg. %N	Avg. δ^15^N
Island area	1.00	0.22	−0.13	0.11	−0.25	0.37	−0.08	−0.26	−0.59
Dist. to shore	0.22	1.00	−0.02	0.04	−0.23	0.18	−0.32	−0.07	−0.19
Dist. to landmass	−0.13	−0.02	1.00	−0.04	−0.04	−0.04	0.00	0.07	0.20
Wrack biomass	0.11	0.04	−0.04	1.00	0.02	0.14	−0.14	0.00	−0.13
Forest openness	−0.25	−0.23	−0.04	0.02	1.00	0.01	0.07	0.11	0.06
Soil moisture	0.37	0.18	−0.04	0.14	0.01	1.00	−0.05	−0.07	−0.32
Plot slope	−0.08	−0.32	0.00	−0.14	0.07	−0.05	1.00	−0.09	0.02
Avg. %N	−0.26	−0.07	0.07	0.00	0.11	−0.07	−0.09	1.00	0.37
Avg. δ^15^N	−0.59	−0.19	0.20	−0.13	0.06	−0.32	0.02	0.37	1.00

*Note*: Island area and distance to landmass are measured at the island level. Wrack biomass is the average wrack biomass in quadrats nearest to the surveyed plot, and average soil %N and δ^15^N are averages of soil samples taken from the 0 m and 40 m plots in each transect. The rest of the parameters were measured in each plot.

**TABLE A4:** Root mean square errors (RMSE) and proportion of variance explained (*R* ^2^) for each species included in a spatially explicit joint species distribution model (JSDM)

Latin name	Explanatory RMSE	Explanatory *R* ^2^	Predictive RMSE	Predictive *R* ^2^
*Blechnum spicant*	.32	.37	.35	.18
*Calamagrostis nutkatensis*	.21	.69	.33	.06
*Conioselinum gmelinii*	.16	.62	.22	.17
*Cornus unalaschkensis*	.24	.71	.35	.30
*Gaultheria shallon*	.19	.66	.26	.33
Lichen	.32	.22	.35	.05
*Linnaea borealis*	.19	.50	.22	.33
*Neottia cordata*	.27	.15	.29	.03
*Maianthemum dilatatum*	.36	.47	.42	.28
*Menziesia ferruginea*	.38	.34	.44	.08
Moss	.25	.47	.30	.05
*Picea sitchensis*	.20	.21	.22	.01
*Platanthera* spp.	.22	.23	.24	.07
*Prenanthes alata*	.24	.57	.34	.05
*Streptopus amplexifolius*	.20	.35	.23	.08
*Thuja plicata*	.27	.36	.32	.04
*Tsuga heterophylla*	.26	.53	.33	.10
*Vaccinium parvifolium*	.35	.42	.42	.12

*Note*: We calculated explanatory RMSE and *R* ^2^ by simulating the posterior predictive distribution using the same data that were used to fit the model. To evaluate predictive power, we conducted a four‐fold cross‐validation, where samples were randomly divided among four partitions. The model is then fit separately for each partition. Here, we also report cross‐validation‐based predictive RMSE and *R* ^2^ values for each species. The predictive power was worse than the explanatory power for each species. All calculations were done using the *Hmsc* package in R v. 4.1.1. (R Core Team, 2021; Tikhonov, Opedal, et al., 2020).

Of the 100 species detected in our surveys, we removed those present in <5% of plots (Stark et al., 2020) when fitting our joint species distribution model. We retained detections of 6141 plants in 1326 plots belonging to 18 species. Of these detections, “moss” and *Gaultheria shallon* were by far the most prevalent—each was found in ~92% of all plots. The next most prevalent species was *Maianthemum dilatatum*, found in 58% of plots. Our model performed well—the mean *R* ^2^ (representing variance explained in species distributions) was 0.44 across species; however, there was some variability in model fit for individual species. The poorest fit was for *Neottia cordata* with an *R* ^2^ of 0.15, but it fit best for *Cornus unalaschkensis*, with an *R* ^2^ of 0.71.
