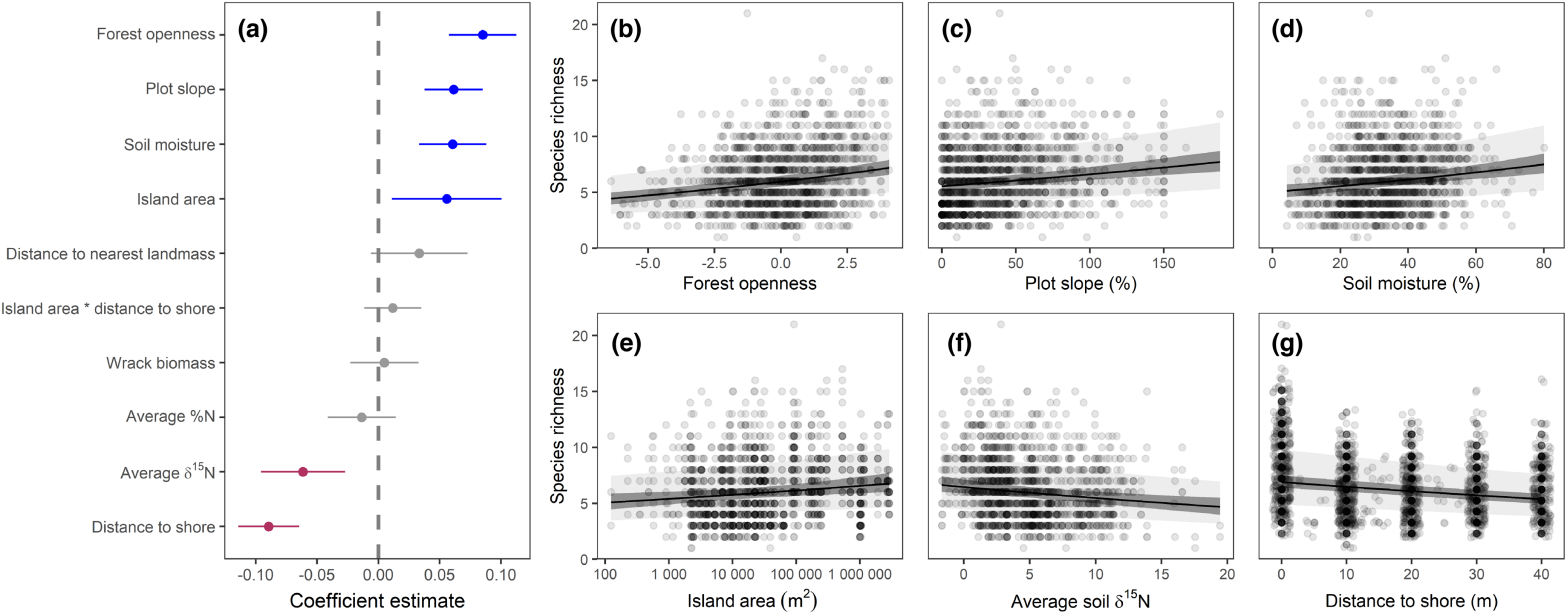# Corrigendum

**DOI:** 10.1002/ece3.9413

**Published:** 2022-10-17

In the recent article by Obrist et al. (2022), the labeling of the y‐axes showing species richness in Figure 3 was unclear. The correct figure is shown below:

**FIGURE 3:**